# A Randomized, Phase II Study of Preoperative plus Postoperative Imatinib in GIST: Evidence of Rapid Radiographic Response and Temporal Induction of Tumor Cell Apoptosis

**DOI:** 10.1245/s10434-008-0177-7

**Published:** 2008-10-25

**Authors:** John C. McAuliffe, Kelly K. Hunt, Alexander J. F. Lazar, Haesun Choi, Wei Qiao, Peter Thall, Raphael E. Pollock, Robert S. Benjamin, Jonathan C. Trent

**Affiliations:** 1 MD/PhD Program, The University of Texas-Houston, Houston, TX, USA; 2 Department of Sarcoma Medical Oncology, The University of Texas M. D. Anderson Cancer Center, 1515 Holcombe Blvd., Suite FC11.3032, Houston, TX 77030, USA; 3 Sarcoma Research Center, The University of Texas M. D. Anderson Cancer Center, Houston, TX, USA; 4 Department of Surgical Oncology, The University of Texas M. D. Anderson Cancer Center, Houston, TX, USA; 5 Department of Pathology, The University of Texas M. D. Anderson Cancer Center, Houston TX, USA; 6 Department of Diagnostic Radiology, The University of Texas M. D. Anderson Cancer Center, Houston, TX, USA; 7 Department of Biostatistics, The University of Texas M. D. Anderson Cancer Center, Houston, TX, USA; 8 Department of Sarcoma Medical Oncology, The University of Texas M. D. Anderson Cancer Center, 1515 Holcombe Blvd., Houston, TX 77030, USA

## Abstract

Gastrointestinal stromal tumor (GIST) is the most common sarcoma arising in the gastrointestinal (GI) tract. Imatinib mesylate (imatinib) is efficacious in treating advanced and metastatic GIST. Patients undergoing resection of GIST realize a highly variable median disease-free survival (DFS). In the absence of prospective data, we conducted a randomized, phase II study to assess the safety and efficacy of preoperative and postoperative imatinib for the treatment of GIST. Nineteen GIST patients undergoing surgical resection were randomized to receive 3, 5, or 7 days of preoperative imatinib (600 mg daily). Patients received postoperative imatinib for 2 years. Perioperative adverse events were compared with those in an imatinib-naïve historical control. The efficacy of imatinib was assessed by ^18^fluorodeoxyglucose positron emission tomography (^18^FDG-PET), dynamic computed tomography (dCT), terminal deoxynucleotidyl transferase dUTP nick end labeling (TUNEL) assay, and DFS. Imatinib did not affect surgical morbidity as compared with an imatinib-naïve cohort (*p* ≥ 0.1). Most patients responded to preoperative imatinib by ^18^FDG-PET and dCT (69% and 71%, respectively). Tumor cell apoptosis increased by an average of 12% (range 0–33%) and correlated with the duration of preoperative imatinib (*p* = 0.04). Median DFS of patients treated with surgery and imatinib was 46 months (range 10–46 months). Tumor size was a predictor of recurrence after postoperative imatinib (*p* = 0.02). Imatinib appears to be safe and may be considered for patients undergoing surgical resection of their GIST. Radiographic response and tumor cell apoptosis occur within the first week of imatinib therapy.

Gastrointestinal stromal tumor (GIST) is the most common sarcoma arising in the GI tract and shares features with the interstitial cell of Cajal, the pacemaker cell of the GI tract.^1,2^ Most GISTs encode activating mutations in either the kit or platelet-derived growth factor receptor-α (PDGFR-α) gene, an important genetic event in tumorigenesis.^3,4^

The use of imatinib mesylate (imatinib; Gleevec™ Novartis, Basel, Switzerland) has revolutionized the management of advanced GIST. Imatinib inhibits the kinase activity of kit, PDGFR-α, and the breakpoint cluster region (BCR)/Abelson (ABL) fusion protein.^5^ Median overall survival for patients with advanced GIST treated with imatinib was longer than 57 months, compared with 9 months for doxorubicin-treated, historical controls.^6,7^

Approximately 46% of all GIST patients are surgical candidates, but 40–90% suffer recurrence within 24 months of complete resection with median disease-free survival (DFS) as low as 18 months. ^8–12^ Thus, a substantial subset of patients may benefit from combined surgery and imatinib. Prospective data on the safety and efficacy of preoperative, perioperative, and postoperative imatinib remain limited. Additionally, there are no prospective studies demonstrating the efficacy of imatinib during the first week of therapy, nor studies to determine whether imatinib's mechanism of action involves tumor cell apoptosis.

Herein, we present the first randomized, phase II study of preoperative and postoperative imatinib for patients with GIST undergoing surgical resection. The objectives of this study were to determine the safety and efficacy of imatinib administered for 3, 5, or 7 days prior to surgery and continued for 24 months postoperatively as well as to determine whether the antitumor activity of imatinib was associated with induction of tumor cell apoptosis.

## Patients and Methods

Patients eligible for this study had histologically proven diagnosis of kit-expressing GIST ≥ 1 cm in greatest diameter never treated with any previous chemotherapy including imatinib for which surgical resection was recommended by an experienced sarcoma surgeon (K.K.H. and R.E.P.). Informed consent was obtained from all patients under a protocol approved by an institutional review board (IRB).

Upon accrual, patients were randomized to receive 3, 5, or 7 days of imatinib (300 mg twice daily by mouth) preoperatively, with the last dose of imatinib given the morning of surgery. Patients restarted imatinib therapy postoperatively and continued it for 2 years at the 600 mg daily dosage (the highest dosage of imatinib approved by the United States Food and Drug Administration). Patients were assessed for adverse events using the Common Terminology Criteria for Adverse Events version 3.0.^13^

This trial was designed for 24 patients in order to achieve the probability of a 2-year disease-free survival reaching an acceptable posterior 90% credible interval. Due to slow accrual, 19 patients were evaluated for outcomes. Patients were randomized in a blinded fashion by our institutional department of biostatistics using an IRB-approved schema (P.T.).

The primary endpoint of this study was tumor cell apoptosis. The secondary endpoints were patient safety, DFS, and radiographic response.

### Retrospective Imatinib-Naïve Cohort

In order to better characterize surgical morbidity in this patient cohort, we collected, and compared, data from a retrospective series of GIST patients treated at our institution undergoing complete resection of their GIST who were not treated with preoperative nor postoperative imatinib. Surgical parameters including estimated blood loss, perioperative complications, and stage of disease were compared with the patients treated on the current prospective study.

### Pre-Imatinib Biopsy and Surgical Resection

All patients were requested to undergo an image-guided [ultrasound or computed tomography (CT)] core needle or endoscopic biopsy of the radiographically viable portion of tumor within 4 weeks prior to initiation of imatinib treatment. Once completing the allocated duration of preoperative imatinib, patients underwent exploratory laparotomy and maximal resection with the attempt to remove all gross disease. Viable tissue was processed rapidly by formalin fixation or Optimal Cutting Temperature compound (OCT; Electron Microscopy Sciences, Hatfield, PA, USA). Disease stage was assessed by patient history and extent of disease as evidenced by radiographic imaging.

### GIST Genotyping

Tumor tissue was assayed for kit and PDGFR-*α* mutation as previously described.^4,14^ Genomic DNA samples were isolated from paraffin-embedded or frozen tissue, polymerase chain reaction was performed, and mutations were identified by a 3730 × 1 DNA Analyzer (Applied Biosystems, Foster City, CA) at MDACC Nucleic Acid Core Facility.

### ^18^Fluorodeoxyglucose Positron Emission Tomography (^18^FDG PET)

Patients underwent ^18^FDG PET imaging before and after preoperative imatinib therapy using a CTI HR + PET scanner (Siemens inc., Knoxville, TN) following administration of 10–15 mCi ^18^FDG over 1 min as described previously.^15^ Scans performed during preoperative imatinib were obtained within 24 h of surgical resection.

Briefly, patients underwent a 60-min uptake phase, a 5-min emission scan, and a 3-min transmission scan per field of view in a two-dimensional mode. Images were interpreted using volumetric and multiple orthogonal projection analysis then quantitated using vendor specific software. Maximal standardized uptake value (SUV_max_) was measured from a region of interest (ROI) representative of tumor on pretreatment images and corresponding ROIs on posttreatment images. Since there is no standardized criteria for PET response at 3, 5, or 7 days after initiation of imatinib for GIST, PET response was defined as either a residual SUV_max_ ≤ 3.9 in post-imatinib scans or a relative 40% decrease in SUV_max_ between pre and post-imatinib scans.^15^

### Dynamic (Perfusion) Computed Tomography (dCT)

dCT was performed before and after preoperative imatinib (within 24 h of surgical resection). All image data were acquired on a Light-Speed or Hi-Speed Advantage helical scanner with multidetector rows (GE Medical Systems, Milwaukee, WI, USA). Fifty milliliters nonionic contrast material (Optiray 320; Mallinckrodt Inc., St. Louis, MO) was injected at a rate of 5 mL/s through an 18-gauge needle in the antecubital fossa. Four 5-mm-thickness slices were obtained through the ROIs with a 5–10 s delay and 30–40 s scan time in CINE mode. Imaging data were postprocessed at an Advanced Workstation (AW HE Healthcare) to calculate the perfusion parameters (blood flow: mL/min/100 g tissue) for all four cross-sections. The mean values of blood flow from all four cross-sections were calculated for response evaluation. dCT response was defined as a decrease in tumoral blood flow ≥ 10%.

### TUNEL Assay

Frozen tissue from biopsy and surgical specimens was assayed for apoptosis using the DeadEnd Fluorometric TUNEL system (Promega Corporation, Madison, WI, USA). Using Spot Advanced for Windows and Image ProPLUS version 6.1 for Windows software, five random fields of view at 200 × magnification were visualized, and cells were counted to determine the percentage of TUNEL-positive nuclei. The average percentage of TUNEL-positive cells in biopsy and surgical tissues were compared.

### Statistical Analyses

Cox survival regression models were used to assess the effects of tumor size, patient age, and sex on DFS.^16^ Univariate Cox survival regression models were used to assess the individual effects of changes in ^18^FDG PET, dCT, and apoptosis on DFS.^16^ Kruskal–Wallis tests were used to determine between-group differences in dCT, ^18^FDG PET, and apoptosis. Martingale residual plots were employed to examine possible functional forms of continuous variables in the fitted Cox models. Owing to the limited sample size (six cases of disease progression in 19 patients), a multivariate Cox model was not fit. Linear regression was used to determine the correlation between dCT and/or ^18^FDG PET and apoptosis response. Unadjusted DFS time probabilities were estimated using the method of Kaplan and Meier.^17^ Survival plots and actuarial life tables were constructed using SPSS version 11.0 for Mac OS X. An extended Cox model with time-varying covariates was employed to examine the effect of imatinib on DFS. Covariate distributions for this cohort and the retrospective patients were compared using generalized Fisher exact tests for categorical variables and Wilcoxon tests for numerical valued variables.^18^

### Role of Funding Source

No funding source (including Novartis) had input in the collection, analysis, and interpretation of data. Novartis did review this manuscript and concurred with the decision to submit this paper for publication.

## Results

### Patient Characteristics Including Tumor Mutation Status

Nineteen patients were accrued from August 8, 2003, to March 26, 2007, at MDACC, TX, USA. The trial schematic is illustrated in Supplemental Data 1. The baseline patient characteristics are provided in Table 1.

Baseline core needle biopsies of tumor were collected prior to imatinib treatment from 14 (74%) of 19 patients. We did not acquire tumor tissue by core-needle biopsy at baseline from five patients owing to lack of consent or untoward risk of biopsy.

Mutation analyses revealed 18 (95%) patients had tumor harboring mutations in either kit or PDGFR-α (Table 1). Interestingly, one patient had both a kit exon 11 duplication and a PDGFR-α exon 12 point mutation. The most common type of mutation was deletion (47% of patients, Supplemental Data 2).

### Surgical Intervention and Perioperative Morbidity

Seventeen of 19 patients underwent a timely resection of their GIST after receiving imatinib. In one patient, the surgical resection was delayed by 3 weeks owing to imatinib-associated toxicity. One patient withdrew consent prior to surgical resection for personal reasons not related to the trial.

The surgical procedures are summarized in Table 2A. All patients had exploratory laparotomy and en bloc resection of tumor and pseudo-capsule, with resection of any gross metastatic deposits.

There were no cases of intraoperative tumor rupture or hemorrhage. Additionally, we observed no episodes of wound dehiscence or delayed healing. One patient had a pelvic abscess that was drained percutaneously and resolved on antibiotic therapy. Three (18%) of 17 patients required brief (<3 days) care in the intensive care unit: need for serum glucose monitoring secondary to type I diabetes mellitus, a non-Q-wave myocardial ischemic event on postoperative day 1, and hyperglycemia secondary to type II diabetes mellitus. Patients began postoperative imatinib a median of 22 days (range 7–59 days) after tumor resection.

As compared with a retrospective analysis of 27 imatinib-naïve patients undergoing surgical resection, similar mean estimated blood loss (605.8 mL, *p* = 0.58), transfusion rates (*p* = 1.0), and perioperative complication rates (*p* = 0.1) were demonstrated (Table 2B).

### Medical Intervention and Adverse Events

The toxicity profile of imatinib in this clinical trial was similar to that in patients with metastatic GIST (Table 3).^6,19^ Most patients tolerated therapy well, and there were no deaths. Five grade 4 events occurred in four (22%) patients. Perioperative vascular events were infrequent (12%, 2 of 17 patients), but one patient had non-Q-wave myocardial ischemia and one patient had a transient ischemic event. Both patients had known, pre-existing vascular disease and both fully recovered to baseline function prior to discharge from the hospital. One patient had grade 4 anemia postoperatively secondary to endometriosis and uterine bleeding.

### Radiographic Efficacy of Preoperative Imatinib

**Fig. 1:**
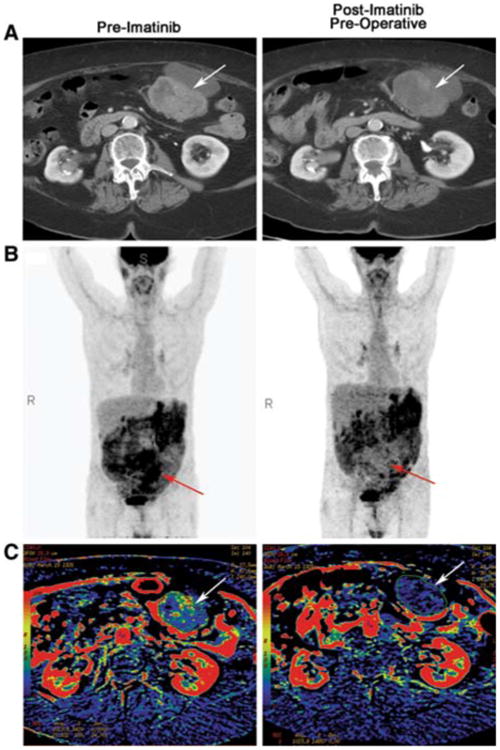
Representative radiographic and functional imaging of GIST (arrows). (**a**) contrast-enhanced CT before and after preoperative imatinib. Some hypodensity within the tumor can be appreciated in presurgical scans compared with baseline scans. (**b**) ^18^FDG-PET before and after preoperative imatinib. Black indicates sites of ^18^FDG accumulation. Complete abrogation of avid disease can be appreciated in this representative patient in latter scans. (**c**) dCT blood flow reconstruction before and after preoperative imatinib. Red indicates blood flow similar to the abdominal aorta; other colors indicate blood flow less than the abdominal aorta, with blue and black representing the least blood flow. A decrease in the amount of red within the tumor in latter scans compared to pre-imatinib scans indicates a decrease in tumor blood flow in response to imatinib

Sixteen (84%) of 19 patients were assessable for response by ^18^FDG PET (Fig. 1). One patient did not complete the imaging studies owing to scheduling errors. Two patients had tumors that did not demonstrate glucose uptake on baseline ^18^FDG PET. Eleven (69%) of 16 patients had tumors that demonstrated ≥ 40% decrease in SUV_max_. Moreover, 10 (63%) of 16 patients had tumors with residual SUV_max_ ≤ 3.9.

Seventeen (89%) of 19 patients had GISTs that were assessable by dCT (Fig. 1). Two patients did not complete the imaging studies because of scheduling errors. Of these 17 patients, 12 (71%) had a >10% (range 11.53–75.14%) decrease in blood flow in the viable regions of their tumors.

Collectively, all patients that were assessed for radiographic response responded to preoperative imatinib by one or more of our criteria. Interestingly, 62% of patients responded exclusively by either a decrease in tumor cell glucose metabolism (PET) or by a decrease in tumor blood flow (dCT) but not both, whereas only 38% responded by both modalities. Thus, we next sought to determine the early cellular effects of imatinib.

### Antitumor Efficacy of Preoperative Imatinib

**Fig. 2:**
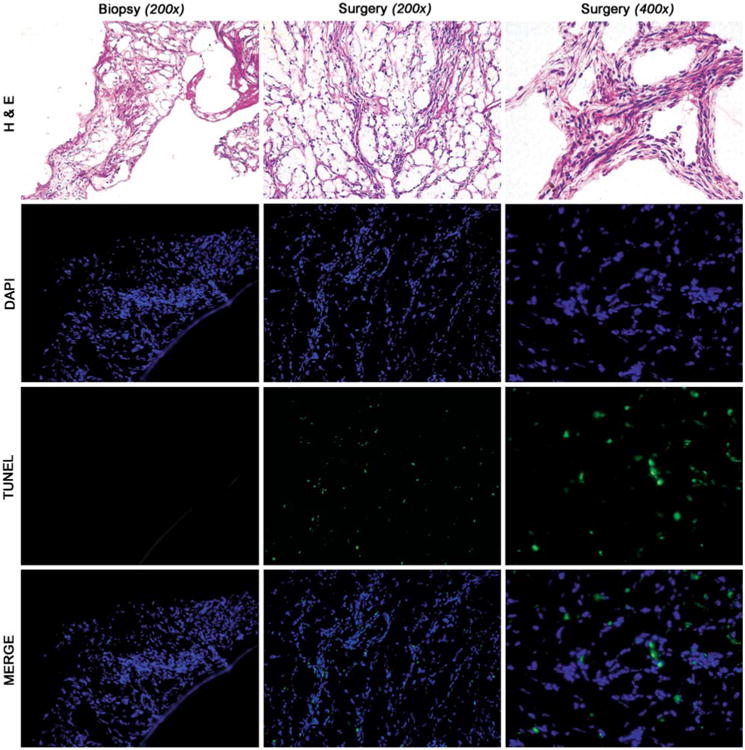
Histologic evaluation of tumor tissue and TUNEL assay. Matched hematoxylin and eosin (H&E) and immunofluorescence of a representative frozen biopsy and surgical specimen from a single patient treated for 5 days with preoperative imatinib. Hypercellular, hyperchromic tissue can be appreciated in both the biopsy and surgical specimens. TUNEL assay indicates that tumor cells underwent apoptosis after 5 days of imatinib: blue, nuclei stained with DAPI; green, fluorescein isothiocyanate (FITC) tag of positive TUNEL reaction; merge, overlay of blue and green indicating nuclei undergoing apoptosis

To better understand the tumoral events leading to the radiographic responses evidenced by ^18^FDG PET and dCT, we comparatively analyzed matched baseline core-needle biopsies and surgical specimens using light microscopy (Fig. 2).

Histologically, equivalently cellular tumor tissue was seen at both baseline and surgery without evidence of myxoid degeneration, a feature characteristic of more prolonged treatment with imatinib. Thus, pathologic response and cytoreduction after this short interval of preoperative imatinib was not appreciated by histological examination in any of the surgical tissues.

We hypothesized that a potential mechanism of initiation of myxoid degeneration was tumor apoptosis. Therefore, we assessed matched pre and post-imatinib tumor tissue for TUNEL. Ten of 14 pretreatment biopsies (4 biopsy specimens were inadequate) and 17 of 17 surgical tumor specimens were analyzed for apoptosis (Fig. 2). Rare TUNEL-positive cells were seen in the pre-imatinib tissue. However, numerous TUNEL-positive cells were observed in the post-imatinib surgical specimens with an absolute increase of 12% for matched tissues (Table 4A).

Moreover, the rate of tumor cell apoptosis was found to be dependent on the duration of preoperative imatinib therapy (Table 4B). Tumor cell apoptosis increased incrementally with duration of imatinib, where patients treated for 7 days with preoperative imatinib had the greatest rate of tumor cell apoptosis (15%, *p* = 0.04).

A high rate of TUNEL-positive cells in the resected tumor specimens tended to be associated with dCT response; however, this trend did not reach statistical significance (*p* = 0.13). Interestingly, PET response by either response criteria had no association with GIST cell apoptosis.

Patients with tumors harboring kit exon 11 mutations had a 14% increase in tumor cell apoptosis after preoperative imatinib (*p* = 0.03) (Table 4C). Conversely, the one patient with tumor harboring a kit exon 9 mutation had no increase in apoptosis with imatinib therapy of 600 mg daily.

### Disease-Free Survival

At the time of this analysis, eight (42%) patients have successfully completed 2 years of postoperative imatinib. Three (16%) patients continue to take postoperative imatinib on the study. Eight (42%) patients discontinued imatinib prior to completing 2 years of postoperative imatinib: four owing to toxicity, two due to withdrawal of consent, and two being lost to follow-up.

**Fig. 3:**
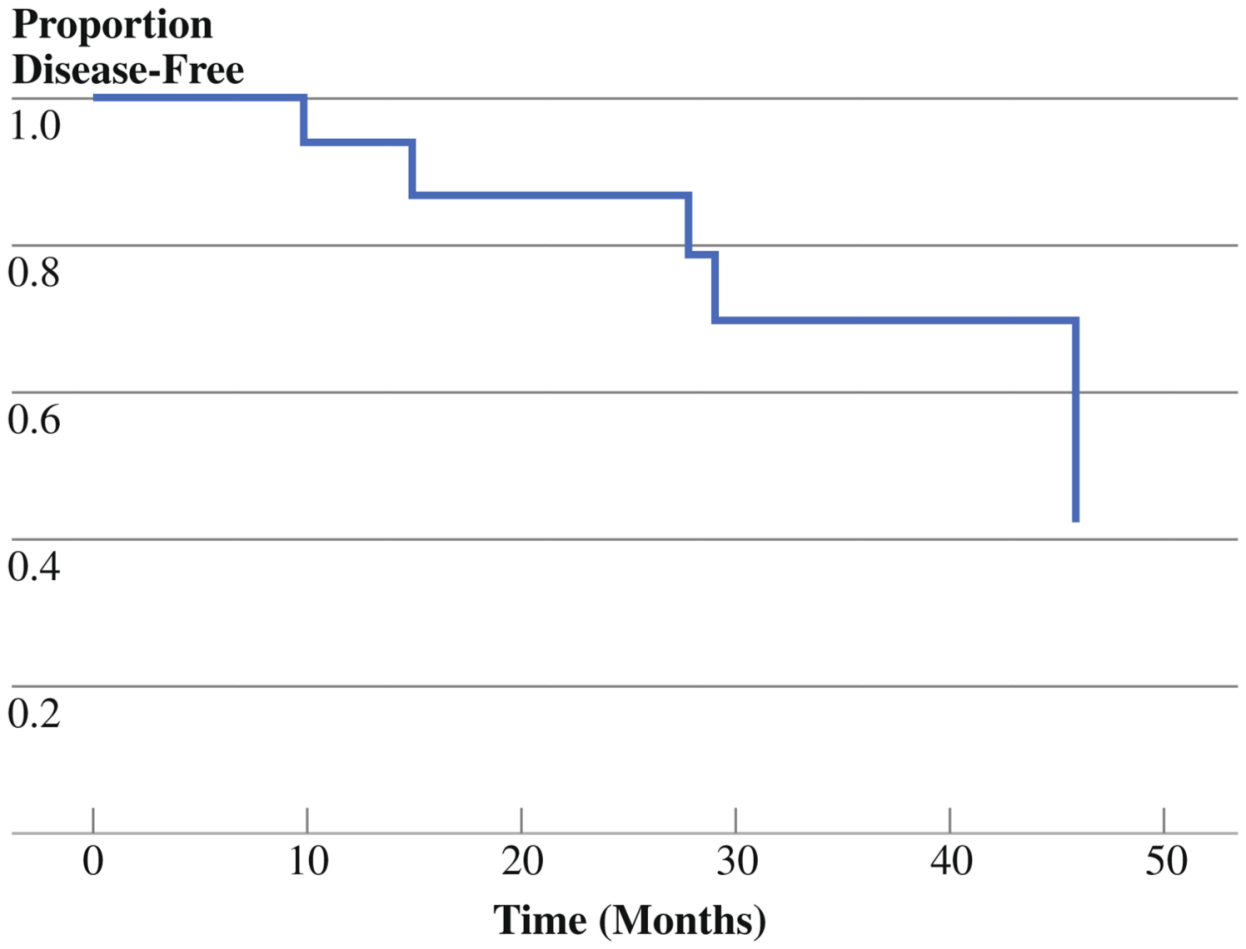
Disease-free survival. Kaplan–Meier plot of DFS versus time (months) since recruitment to trial. Median DFS = 46 months. Tick marks = patients censored

With median follow-up of 32 months, median duration of DFS was 46 months (Fig. 3). Actuarial DFS rates were 94% and 87% at 1 and 2 years, respectively. Six (32%) of the 19 patients have had a recurrence, but no patients have died. Randomization to 3, 5, or 7 days of preoperative imatinib did not affect DFS (*p* = 0.71).

Interestingly, all patients who had a recurrence on the study had disease that originated in the small bowel. Moreover, these patients either had a large tumor burden (>10 cm) or presented with metastatic and/or recurrent disease. Not surprisingly, larger tumor size predicted shorter DFS (*p* = 0.02). There was a trend between longer DFS and response measured by dCT and higher levels of tumor apoptosis, but neither reached statistical significance. Moreover, PET response was not predictive of DFS duration.

All six patients who had tumor recurrence had stopped taking imatinib due to completion of the study, noncompliance, or toxicity at the time of the recurrence. Five of these six patients harbored kit exon 11 mutation and were treated for 24, 24, 24, 8, and 15 months postoperatively. The other patient harbored a kit exon 9 mutation and was treated for 24 months postoperatively. Median time to disease progression for these patients after discontinuation of imatinib was 4 months. However, the likelihood of recurrence was not associated with whether a patient was receiving imatinib (*p* = 0.99), accounting for the fact that the actual schedule of receiving or not receiving imatinib over time varied between patients.

No patient recurred at the cutaneous site of tumor biopsy but rather in the peritoneum (three) and at hepatic sites (three). The five patients that restarted imatinib therapy (one of these patients was lost to follow-up) had a subsequent response as assessed by contrast-enhanced CT.

## Discussion

We found that imatinib therapy appears to be safe when given to GIST patients preoperatively, including hours prior to surgical resection. Furthermore, we provide direct evidence that radiographic response can be observed in the first week of therapy and that tumor cell apoptosis increases incrementally with duration of preoperative imatinib therapy. Lastly, we show that postoperative administration of imatinib improved duration of DFS in our GIST patients at 1 and 2 years compared with that in historical controls (ten). Thus, patients undergoing resection of their GISTs may be considered for therapy with imatinib to prolong disease-free survival.

It is our belief that the molecular mechanisms of imatinib efficacy are initiated prior to detectable histopathologic cytoreduction. Thus, we chose to treat patients for 3, 5, or 7 days in the hope of demonstrating time-dependent molecular and/or functional changes without cytoreduction. Our data demonstrate that, within 3–7 days of imatinib therapy, no cytoreduction is appreciable histologically, yet we found no correlation between functional radiographic imaging and apoptosis of tumor cells. These findings suggest the radiographic responses observed when a GIST patient is treated with imatinib are not due entirely to GIST cell death.

Interestingly, the one patient without an increase in tumor cell apoptosis harbored a kit exon 9 mutation and was treated with only 600 mg daily imatinib. This finding suggests the possibility that treating patients harboring exon 9 mutation with 600 mg daily imatinib may be suboptimal as seen in patients with metastatic disease treated with 400 mg daily doses.^20^

As evidenced by dCT, tumor blood flow decreases in most patients within 3–7 days of initiation of imatinib therapy. An antivascular effect of imatinib in GIST may play a role in radiographic responses, surgical outcomes, and perhaps patient survival. Multiple mechanisms explaining this phenomenon have been suggested. Nitric oxide production via nitric oxide synthase may be a downstream effector of kit and abrogated in response to kit inhibition, leading to collapse of the vascular architecture.^21,22^ Alternatively, imatinib may target kit and/or PDGFR signaling in tumor-associated endothelial cells or pericytes.^23–25^

The American College of Surgeons Oncology Group (ACOSOG) is evaluating adjuvant imatinib versus placebo for primary GIST. Preliminary results demonstrate a DFS rate of 97% at 1 year for patients receiving imatinib compared with 83% at 1 year following complete resection in patients receiving placebo.^26^ Although our study included higher-risk patients, we report a similar 1-year DFS rate of 94%, with a 2-year DFS rate of 87%. Although marginal, the higher 2-year DFS rate in our study may be due to treatment of patients with postoperative imatinib for 2 years as compared with only 1 year in the ACOSOG study. Thus, our study is supported by preliminary prospective and retrospective data showing that adjuvant imatinib extends DFS compared with surgery alone.^26,27^ Moreover, our findings corroborate the preliminary data that larger tumor size predicts shorter DFS for patients with a resected GIST treated with imatinib postoperatively.^26^ This implies that patients with large tumor burden (>10 cm in diameter) may benefit from longer duration of adjuvant imatinib.

Importantly, not a single patient in this study had a recurrence while receiving postoperative imatinib therapy. This observation is interesting when considered in the context of a randomized discontinuation study that found patients with metastatic GIST who responded to imatinib therapy had a markedly increased risk of progression upon discontinuation of imatinib.^28^ Moreover, anecdotal studies have found that residual quiescent tumor cells are observed in tumors from GIST patients that have been resected after response to imatinib.^28–30^ Thus, our results suggest that the action of imatinib may be both cytotoxic by evidence of apoptosis and cytostatic as observed in other studies that have found quiescent GIST cells. It is possible that imatinib therapy, even in the adjuvant setting, should be continued until toxicity, recurrence, or progression occurs. Hopefully ongoing and future clinical trials will adequately determine the duration of postoperative imatinib therapy.

Collectively, our study is the first prospective, randomized trial to show the safety and efficacy of combining preoperative with postoperative imatinib. We are the first to report early decreases in both tumor cell glucose metabolism by FDG-PET and tumor blood flow by dCT as well as a time-dependent increase in tumor cell apoptosis after imatinib.

## Supplementary Material

Supplemental Data 1

Supplemental Data 2

## Figures and Tables

**Table 1:** Patient and tumor characteristics

Characteristic		
Age (years)		
Mean (95% confidence)	59	(7)
Tumor size (cm)
Mean (95% confidence)	9	(3)

	No.	%

Gender		
Male	11	58
Female	8	42
Ethnic origin		
White	10	53
Black	5	26
Hispanic	1	5
Asian	3	16
Primary site		
Stomach	13	68
Small bowel	6	32
Disease status		
Primary, no metastasis	13	68
Primary, with metastasis	2	11
Local recurrence, with metastasis	1	5
Recurrence, metastatic	3	16
Randomization (duration of preoperative imatinib)		
3 days	7	36
5 days	6	32
7 days	6	32
GIST genotype		
Kit exon 11 mutation	14	74
Kit exon 9 mutation	1	5
PDGFR-α exon 12 mutation	2	11
Kit exon 11 and PDGFR-α exon 12 mutation	1	5
Wild type^a^	1	5

a Wild type, no mutations found in exons analyzed

**Table 2:** (A) Types of organ resections performed in patients undergoing complete resection of their GISTs after preoperative imatinib. (B) Comparison of surgical characteristics and morbidities in patients treated with or without preoperative imatinib. MDACC ID030023 patients received 3–7 days imatinib prior to surgical resection

(A)					

Characteristic (*n* = 17, MDACC ID03-0023)	No.		%		
Organ resection^a^					
Partial gastrectomy	10		59		
Partial duodenectomy	2		12		
Partial jejunectomy	4		24		
Partial colectomy	4		24		
Partial mesentectomy	3		18		
Partial omentectomy	3		18		
Partial hepatectomy	2		12		
Distal pancreatectomy	2		12		
Splenectomy	2		12		
Adrenalectomy	1		6		
Multi-organ resection	9		53		
Patients requiring transfusion	7		41		
Postoperative ICU monitoring	3		18		

	Mean		Range		

Tumor size (cm)	9		1–23		
EBL (mL)	735		25–3,600		
Transfusion volume (mL)^b^	1,230		500–2,500		
Duration of hospital stay (days)	10		5–15		

(B)					

Characteristic	MDACC ID030023		Imatinib-Naïve (*n* = 27)		*p*-value
	
	Mean	±SD	Mean	±SD	

Tumor size (cm)	9.5	6.3	10.2	6.5	0.81
EBL (mL)	735.3	913.7	605.8	685.3	0.58
	*n*	%	*n*	%	
Disease stage at surgery					
Primary	12	71	17	63	0.75
Metastatic/recurrent	5	29	10	37	
Need for blood transfusion					
No	10	59	17	63	1.0
Yes	7	41	10	37	
Perioperative adverse events					
No	14	82	15	56	0.1
Yes	3	18	12	44	

Imatinib-naïve, retrospective cohort of GIST patients who underwent complete resection without prior imatinib therapy; ICU, intensive care unit; EBL, estimated blood loss; SD, standard deviation

a Nine of 17 patients required multi-organ complete and/or partial resection of adjacent tissues to remove all gross disease. Thus, the total number of organ resections is greater than 17

b Only seven patients required a blood transfusion during surgery

**Table 3:** Grade 3 and 4 adverse events experienced by patients. Adverse events are separated based on treatment phase

Event	Preop.	Periop.	Postop.
Edema			*1*
Nausea/vomiting	**1**		*1*
Supraventricular arrhythmia			*1*
Dehydration	**1**		
Pelvic abscess		*1*	
Stroke		**1**	
Elevated troponin T		**1**	
Hypocalcemia			*1*
Anemia			**1**
Dizziness			*1*
Memory impairment			*1*
Fatigue			*1*
Anorexia			*1*
Vaginal bleeding			*1*
Total grade 3/4 events	*1*	*1*	*10*
Patients w/grade 4 events	*1*	*1*	*1*

Preop., adverse events during 3–7 days of neoadjuvant imatinib; Periop., events during surgery and/or prior to starting postoperative imatinib; Postop., events after starting adjuvant imatinib. Numbers in bold indicate grade 4 adverse events. Numbers in italic indicate grade 3 adverse events

**Table 4:** Summary of TUNEL assay findings. (A) Average amount of TUNEL-positive nuclei found in all patient tumor biopsies (pre-imatinib) and surgical tissue (post-imatinib) and the mean absolute change in apoptosis due to preoperative imatinib. (B) Mean incremental increase in apoptosis with duration of preoperative (Pre-op) imatinib. (C) Change in apoptosis in response to preoperative imatinib based on patient tumor genotype

(A)				

	% Apoptosis (mean)		Range	
Pre-Imatinib (*n* = 10)	2		0–6	
Post-Imatinib (*n* = 16)	11		3–33	
Absolute change (*n* = 10)	12		0–33	

(B)				

Preop. Imatinib (days)	Absolute Δ % apoptosis (mean)			

3	5.6			
5	9			
7	15			
*p*-value	0.04			

(C)				

GIST genotype	No.	Absolute Δ % apoptosis (mean)	Range	*p*-value

kit exon 11	7	14	1–33	0.03
kit exon 9	1	0	NA	NA
PDGFR-*α* exon 12	1	8	NA	NA
kit exon 11 and PDGFR-*α* exon 12	1	11	NA	NA

NA, could not be assessed statistically because of limited sample size; Δ, change
