# Supplementary figures and images for: A Randomized, Phase II Study of Preoperative plus Postoperative Imatinib in GIST: Evidence of Rapid Radiographic Response and Temporal Induction of Tumor Cell Apoptosis
Source: Ann Surg Oncol. Author manuscript; Available in PMC 2017 Oct 19. (PMC5647649; doi:10.1245/s10434-008-0177-7)

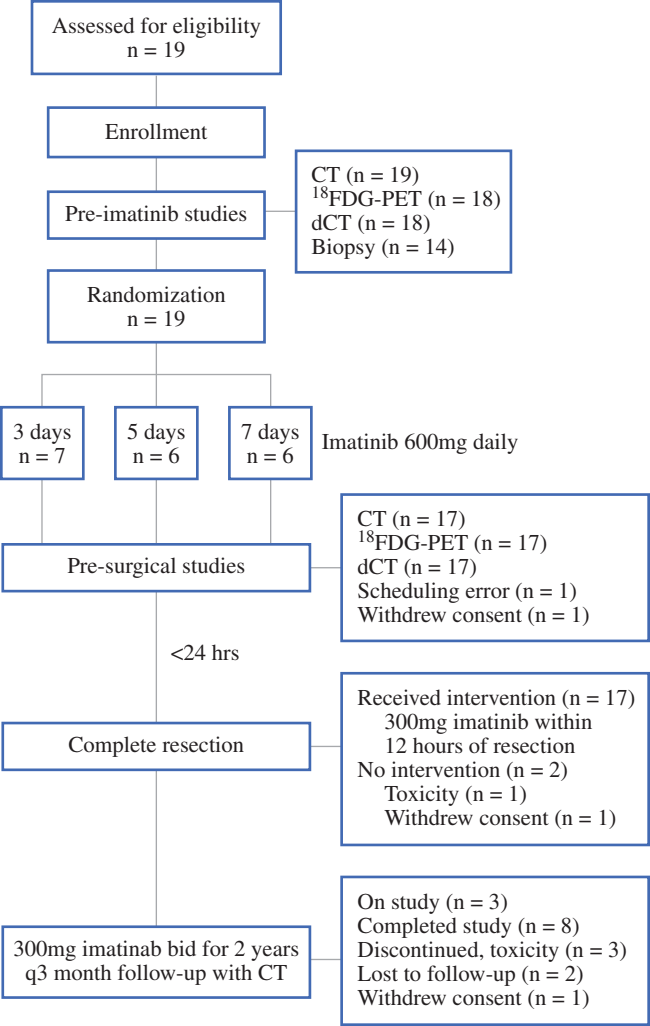

Supplement: Supplemental Data 1 [file NIHMS889433-supplement-Supplemental_Data_1.pdf]
