# Supplementary material for: A Randomized, Phase II Study of Preoperative plus Postoperative Imatinib in GIST: Evidence of Rapid Radiographic Response and Temporal Induction of Tumor Cell Apoptosis
Source: Ann Surg Oncol. Author manuscript; Available in PMC 2017 Oct 19. (PMC5647649; doi:10.1245/s10434-008-0177-7)
Supplement: Supplemental Data 2 [file NIHMS889433-supplement-Supplemental_Data_2.pdf]

Supplemental Data 2. *McAuliffe, et al 2008*

Pt #	Gene	Mutation(s)	Amino Acid
1	*kit* exon 11	point	A567T, L568G
		deletion	Y570_Q575del
2	*kit* exon 11	deletion	W557_V558 del
3	*PDGFR-α* exon 12	point	D583E
4	*kit* exon 11	deletion	V560 del
5	*kit* exon 11	deletion	V555_Q556 del
6	*kit* exon 9	duplication	A502_Y503 dup
7	*kit* exon 11	insertion	P577_H580 dup
8	*kit* exon 11	point	V559D
9	*kit* exon 11	insertion	P577_F591 ins
10	*kit* exon 11	duplication	V575_W577 dup
	PDGFR-α exon 12	point	S478P
11	*kit* exon 11	deletion	V559_Y570 del
12	*kit* exon 11	point	W557R
13	*kit* exon 11	deletion	W557_K558 del
14	none		
15	*kit* exon 11	deletion	W557_K558 del
16	*kit* exon 11	deletion	E554_K558 del
17	*kit* exon 11	point	K558S
		deletion	560V del
18	*kit* exon 11	point	L576P
19	*PDGFR-α* exon 12	deletion	R554 del, Y555S
